# Digitale Lernmethoden in der Pharmazie

**DOI:** 10.1007/s00103-025-04041-5

**Published:** 2025-04-01

**Authors:** Christoph Ritter

**Affiliations:** Institut für Pharmazie, Klinische Pharmazie, Universität Greifswald, Friedrich-Ludwig-Jahn-Str. 17, 17489 Greifswald, Deutschland

**Keywords:** Digitale Lehre, Pharmazie, Deutschland, Anwendungsbeispiele, Künstliche Intelligenz, Digital teaching, Pharmacy, Germany, Application examples, Artificial intelligence

## Abstract

Mit Ausbruch der SARS-CoV-2-Pandemie im März 2020 und den damit verbundenen Beschränkungen im Lehrbetrieb wurden an vielen Hochschulen digitale Lernmethoden verstärkt eingesetzt. Digitale Lernmethoden umfassen im Allgemeinen vollständig oder teilweise digitalisierte Lernelemente wie Vorlesungsaufzeichnungen, freie Lernmaterialien oder E‑Portfolios. Zu den vollständig oder teilweise digitalisierten Lernformaten gehören das Game-based Learning, der Inverted Classroom, mobiles Lernen, die Nutzung sozialer Medien, Online-Peer- und kollaboratives Lernen sowie das adaptive Lernen. Digitalisierte Wirklichkeiten werden im Rahmen von simulationsgestütztem Lernen und in der Augmented und der Virtual Reality erschaffen. Onlinebasierte Veranstaltungsformate und Online-Studiengänge bestehen fast ausschließlich aus internetgestützten Lernphasen.

Inwieweit digitale Lernmethoden in Lehrveranstaltungen der Pharmazie in Deutschland eingesetzt werden, wird in diesem Artikel anhand ausgewählter Praxisbeispiele erläutert. Die ausgewählten Beispiele umfassen die Erstellung eines Audio-Podcasts zur Leistungsbeurteilung einer Praktikumsstation im Praktikum Klinische Chemie als Form eines digitalen Lernelements, die Nutzung eines digitalen Analyseinstruments zur Durchführung von Medikationsanalysen als Beispiel für mobiles Lernen, ein Blended-Learning-Konzept zur Vermittlung von Grundlagen der klinischen Pharmazie, ein Online-Konzept der virtuellen Lehre am Krankenbett und eine spielähnliche Simulation zur Abgabe von Arzneimitteln. Die Einbeziehung von künstlicher Intelligenz kann bei der Entwicklung und Durchführung digitaler Lernangebote hilfreich sein. Dabei müssen jedoch eine ausreichend hohe Qualität und ein kritischer Umgang gewährleistet sein.

## Abstract (en)

With the outbreak of the SARS-CoV‑2 pandemic in March 2020 and the associated restrictions on teaching, digital learning methods were increasingly used at many universities. Digital learning methods generally include fully or partially digitized learning elements such as lecture recordings, open learning materials, or e‑portfolios. Fully or partially digitized learning formats include game-based learning, the inverted classroom, mobile learning, the use of social media, online peer and collaborative learning, and adaptive learning. Digitized realities are created in the context of simulation-based learning and in augmented and virtual reality. Online-based event formats and online degree programs are characterized by an almost exclusive proportion of internet-based learning phases.

The extent to which digital learning methods are used in pharmacy courses in Germany is explained in this article using selected practical examples. The selected examples include the creation of an audio podcast to assess the performance of a clinical chemistry internship as a form of digital learning element, the use of a digital analysis tool to carry out medication analyses as an example of mobile learning, a blended learning concept to teach the basics of clinical pharmacy, an online concept of virtual bedside teaching, and a game-like simulation for dispensing medicines. The inclusion of artificial intelligence can be helpful in the development and implementation of digital learning offerings. However, a sufficiently high quality and critical approach must be guaranteed.

## Einleitung

Mit dem Einzug der technischen Möglichkeiten wurden auch digitale Lernangebote entwickelt. Der Ausbruch der SARS-CoV-2-Pandemie im März 2020 hat den Einsatz digitaler Lernmethoden an Hochschulen gefördert, um den Lehrbetrieb unter den besonderen Bedingungen zur Verringerung der Ausbreitung der Pandemie aufrechterhalten zu können. Dieser Schub hat dazu geführt, dass die während der Pandemie verstärkt entwickelten Lehrangeboten auch nach dem Ende der Pandemie weiter genutzt oder sogar weiterentwickelt werden. Lag der Anteil an reiner Präsenzlehre laut einer bundesweiten Online-Befragung von Hochschulleitungen aus dem Jahr 2021 vor der Pandemie bei durchschnittlich 85 %, wird ein Rückgang auf 59 % erwartet. Ein deutlicher Zuwachs ist insbesondere bei Mischformaten zu erwarten, der Anteil dieser Formate lag vor der Pandemie bei 8 % und wird nach der Pandemie auf 23 % geschätzt. Es wird davon ausgegangen, dass auch der Anteil reiner Online-Formate von 7 % vor der Pandemie auf 18 % ansteigen wird [1].

Die Akzeptanz der digitalen Lehrangebote bei Studierenden ist hoch, so bewerteten im Rahmen des Rankings der Masterstudiengänge 2021 des Centrums für Hochschulentwicklung (CHE) 82 % der befragten Studierenden die Vielfalt der digitalen Lehrangebote als sehr gut oder gut und 89 % der Studierenden bevorzugten für die Zukunft ein Lernsetting, das digitale Lernangebote enthält [2]. Inwieweit digitale Lernangebote gegenüber der reinen Präsenzlehre zu einem höheren Lernerfolg beitragen können, kann pauschal nicht beantwortet werden, da die Bandbreite digitaler Lernangebote sehr hoch ist.

Schon 2016 hat Klaus Wannemacher vom HIS-Institut für Hochschulentwicklung auf der Grundlage von nationalen und internationalen Fallstudien und Fallbeispielen digitalisierte Lernelemente und Lernformate identifiziert und kategorisiert sowie diese zu 8 digitalisierten Lernszenarien aggregiert [3]. Diese Lernelemente, Lernformate und Lernszenarien sollen im Folgenden dargestellt werden. Um zu zeigen, dass diese digitalen Lehr- und Lernmethoden auch in der Pharmazie angewendet werden können, werden ausgewählte digitale Lernangebote von deutschen Hochschulstandorten aus der Pharmazie vorgestellt. Am Ende dieser Übersicht wird diskutiert, wie künstliche Intelligenz bei der Erstellung und Durchführung moderner Lernformate hilfreich sein kann.

## Spektrum digitaler Lernmethoden

### Digitalisierte Lernelemente und -formate

**Abb. 1:**
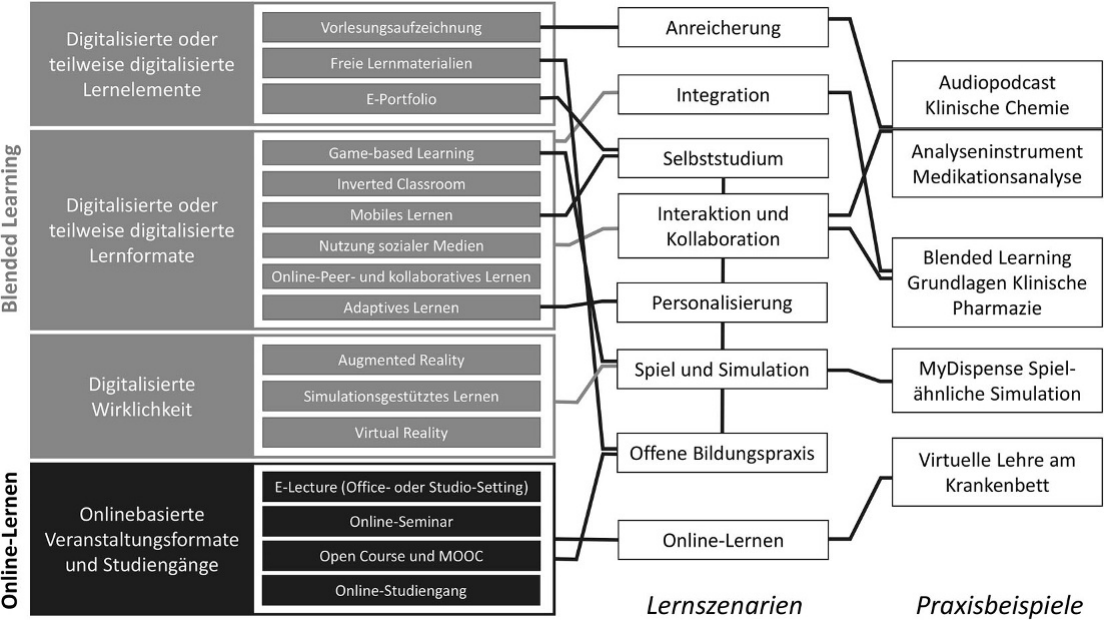
Darstellung der Systematik von Lernelementen, Lernformaten und Lernszenarien und die Einordnung der erläuterten Praxisbeispiele für digitale Lernangebote in der Pharmazie. Abbildung modifiziert nach [3]. Mit freundlicher Genehmigung des Hochschulforums Digitalisierung, Berlin. *MOOC* Massive Open Online Course

Die zur Durchführung digitaler Lernmethoden nutzbaren Lernelemente und Lernformate lassen sich in 4 Kategorien einteilen (Abb. 1). Diese werden im Folgenden beschrieben:

#### Digitalisierte oder teilweise digitalisierte Lernelemente.

Zur Kategorie der digitalisierten oder teilweise digitalisierten Lernelemente gehören die Vorlesungsaufzeichnung, freie Lernmaterialien und das E‑Portfolio. Bei *Vorlesungsaufzeichnungen *handelt es sich um eine digitale Reproduktion und Aufbereitung einer Vorlesung, die Studierenden über eine Website zur Verfügung gestellt werden. Der Lehrende entscheidet, wem die Aufzeichnung zur Verfügung gestellt wird, die Lernenden entscheiden, wann und wie oft sie die Aufzeichnungen aufrufen. *Freie Lernmaterialien* können kostenfrei genutzt, bearbeitet und weiter verbreitet werden. *E‑Portfolios* stellen elektronisch geführte Sammlungen von Dokumenten dar, die den Lernprozess eines Studierenden abbilden sollen. Durch E‑Portfolios kann die Entwicklung eines Studierenden dokumentiert werden, sie können aber auch zur Beurteilung von Lernleistungen herangezogen werden oder sie können der Außendarstellung der durch den Studierenden erworbenen Kompetenzen dienen.

#### Digitalisierte oder teilweise digitalisierte Lernformate.

Während es sich bei Lernelementen um einzelne Lernmaterialien, Lernobjekte oder Software-Anwendungen handelt, stellen Lernformate Lernverfahren, -methoden oder -situationen dar. Digitalisierte oder teilweise digitalisierte Lernformate sind das Game-based Learning, der Inverted Classroom, mobiles Lernen, die Nutzung sozialer Medien, Online-Peer- und kollaboratives Lernen sowie das adaptive Lernen. *Game-based Learning* ist ein Lernkonzept, bei dem der Kompetenzerwerb mit spielerischen Elementen verknüpft wird. Beispiele für Lernspielformen sind Quizze, Simulationen, Educaching, Augmented Reality, gestenbasierte Spiele, Action‑, Abenteuer- und Rollenspiele sowie Massively Multiplayer Online Games. Lernspiele sollen die Lernmotivation der Studierenden erhöhen. Beim *Inverted Classroom* wird die traditionelle Vermittlung von Wissen über Frontalveranstaltungen in Präsenz mit selbstständiger Nachbereitung umgekehrt. Die Studierenden bereiten sich anhand verschiedener digital zur Verfügung gestellter Materialien auf ein Thema vor, das dann in Präsenzveranstaltungen besprochen und vertieft werden kann. *Mobiles Lernen* beschreibt die Möglichkeit, orts- und zeitungebunden mittels eines mobilen Endgerätes Wissensinhalte und Informationen zu recherchieren. Mobiles Lernen kann dabei sowohl im Kontext einer Lehrveranstaltung realisiert werden als auch völlig losgelöst davon. *Soziale Medien* können als digitale Lernformate in verschiedener Weise genutzt werden. Sowohl die Nutzung als digitales Austauschmedium über die verschiedensten Austauschdienste als auch die Nutzung von Plattformen zur gemeinsamen Bearbeitung von Dokumenten zählen zu diesem Lernformat. Beim *Online-Peer- und kollaborativen Lernen *steht der Online-Austausch mehrerer Studierenden im Mittelpunkt. Der Austausch dient dazu, in Seminar- oder Lerngruppen Fragestellungen zu diskutieren und Lösungswege zu erarbeiten. Der Lernprozess besteht bei diesem Lernformat darin, durch den gemeinsamen Austausch von Wissen und Erfahrung ein gemeinsames Verständnis für Sachverhalte zu entwickeln. Dazu gehört zunehmend auch die Möglichkeit, dass Studierende mithilfe von Peer-Grading- oder Peer-Assessment-Instrumenten gegenseitig ihre Leistungen bewerten. Um *adaptives Lernen* zu ermöglichen, muss die Lernumgebung an das individuelle Lernbedürfnis jedes einzelnen Studierenden angepasst werden. Dies kann zum Beispiel durch das Anlegen von Lernpfaden realisiert werden, in denen der Lernfortschritt schrittweise anhand von Wissensfragen ermittelt wird und anhand der Ergebnisse die entsprechenden Lernmaterialien zur Verfügung gestellt werden. Eine spezielle Form ist die adaptierbare Lernumgebung, in der ein Studierender selbst Anpassungen der Lernumgebung vornehmen kann.

#### Digitalisierte Wirklichkeit.

In diese Kategorie der Lernformate fallen Augmented Reality, simulationsgestütztes Lernen und Virtual Reality. Bei Formaten der *Augmented Reality* werden in die reale Lernumgebung ergänzende Informationen visuell eingeblendet. Dies kann durch Smartphones, Tablets oder spezielle Augmented-Reality-Brillen realisiert werden. Um *simulationsgestütztes Lernen* zu ermöglichen, werden bestimmte Situationen und Umgebungen durch interaktive Visualisierung simuliert. Die Studierenden können so Prozesse erkunden und durch Veränderung von Parametern Beziehungen zwischen Ursachen und Wirkungen erfahren. Ein Beispiel ist die Simulationssoftware TDMx, mit der der Konzentrations-Zeit-Verlauf von Arzneistoffen im menschlichen Blut in Abhängigkeit verschiedener Patienten-spezifischer Eigenschaften simuliert werden kann [4]. Zur Realisierung von *Virtual Reality* sind 3‑D-Simulations- oder Grafik-Software sowie spezielle Eingabe- (z. B. 3‑D-Maus, Datenhandschuh) und Ausgabegeräte (z. B. Virtual-Reality-Brille) notwendig. Mittels Virtual Reality können theoretische oder prozessuale Zusammenhänge erfasst, praktische Kenntnisse vermittelt und Fähigkeiten trainiert werden. Dazu können Explorations‑, Trainings‑, Experimental- oder Konstruktionswelten genutzt werden.

#### Onlinebasierte Veranstaltungsformate und Studiengänge.

Onlinebasierte Veranstaltungsformate und Studiengänge sind dadurch gekennzeichnet, dass der Anteil an internetgestützten Lernphasen mindestens 80 % beträgt. Zu dieser Kategorie zählen die E‑Lecture (Office- oder Studio-Setting), das Online-Seminar, der Open Course und der Online-Studiengang. Die *E‑Lecture *wird im Gegensatz zur Vorlesungsaufzeichnung ohne Publikum und ohne korrespondierende Präsenzveranstaltung aufgezeichnet. Das *Online-Seminar* wird vollständig über Webkonferenzdienste umgesetzt. *Open Courses *basieren auf dem Prinzip des freien Zugangs und unbegrenzter Teilnehmer. In Massive Open Online Courses (MOOC) werden verschiedene Formen der Wissensvermittlung kombiniert. Es können Lernvideos, Foliensätze, Foren, Übungsaufgaben und Seminartexte verwendet werden. In diesem Lernformat können sehr hohe Teilnehmerzahlen erreicht werden. In *Online-Studiengängen* wird nahezu das gesamte Curriculum eines Studiengangs online absolviert.

### Digitalisierte Lernszenarien

Die oben beschriebenen Lernelemente und -formate wurden von Wannemacher und Kollegen zu 8 digitalisierten Lernszenarien verdichtet ([3]; Abb. 1). Bei der Aggregation wurden Szenarien gebildet, die sich in verschiedenen Merkmalen, wie z. B. dem Grad der Virtualisierung, der Interaktion oder der Individualisierung, weitgehend unterscheiden, während die Lernelemente und -formate innerhalb der Szenarien möglichst ähnlich sein sollten.

Die im Folgenden dargestellten Lernszenarien schließen sich in der Anwendung gegenseitig aus, da sie Lernszenarien mit unterschiedlichem Grad der Nutzung digitalisierter Lernformate darstellen: Beim Lernszenario *Anreicherung *werden klassische Präsenzveranstaltungen durch digitale Elemente ergänzt. Der Präsenzcharakter und die Rollenverteilung zwischen Lehrenden und Lernenden werden durch dieses Lernszenario nicht verändert. Das Lernszenario *Integration* bildet Konzepte des Blended Learning ab, bei dem traditionelle Präsenzveranstaltungen mit E‑Learning verknüpft werden. Charakteristisch sind hierbei sich abwechselnde, aufeinander abgestimmte Lernphasen mit Präsenzveranstaltungen und digitalen Lernformaten. Auch das Konzept des Inverted Classroom ist diesem Lernszenario zuzuordnen. Beim Lernszenario *Online-Lernen* werden nahezu alle oder alle Lehrveranstaltungen digital abgehalten. Lernformate in diesem Szenario sind E‑Lectures und Online-Seminare, es zählen dazu aber auch ganze Online-Studiengänge.

Dagegen können diese Lernszenarien miteinander kombiniert werden: Beim Lernszenario *Interaktion und Kollaboration* steht das gemeinsame Lernen im Vordergrund. Von zentraler Bedeutung sind hier Instrumente des Austauschs zwischen den Lernenden. Daher werden diesem Lernszenario alle Formen der Nutzung sozialer Medien und Austauschplattformen zugeordnet. Die Rolle des Lehrenden wechselt in diesem Lernszenario vom reinen Wissensvermittler zum Begleiter und Moderator des Lernprozesses. Kernpunkte des Lernszenarios *offene Bildungspraxis* sind der freie Zugang zu Lernmaterialien und der kollaborative Austausch zwischen den Lernenden. In diesem Lernszenario werden typischerweise Open-Course-Lernformate verwendet, es können in dieses Lernszenario aber auch ganze Online-Studiengänge fallen. Das Lernszenario *Spiel und Simulation* basiert auf einer Vermittlung und Aneignung von Lerninhalten mithilfe spielerischer Elemente. Dazu gehören alle Formate des Game-based Learning sowie simulationsgestützte Lernformen und Augmented und Virtual Reality. Beim Lernszenario *Personalisierung* steht die Anpassung an individuelle Lernbedürfnisse und Lernfortschritte im Mittelpunkt. Alle Lernformate des adaptiven oder adaptierbaren Lernens können hier zur Anwendung kommen. Im Lernszenario *Selbststudium* werde alle Lernformen und -formate zusammengefasst, die im Rahmen von Präsenzveranstaltungen Prozesse des Selbststudiums digitalisiert unterstützen. Dazu zählen Formate des mobilen Lernens ebenso wie E‑Assessments und E‑Portfolios.

Diese Übersicht des Spektrums digitaler Lernmethoden zeigt, dass deren Nutzung auf vielfältige Art und Weise möglich ist.

## Digitale Lernmethoden in der Pharmazie

Da das Studium der Pharmazie in Deutschland sehr stark durch praktische Lehrveranstaltungen geprägt ist, wurde an vielen Hochschulstandorten auch während der Pandemie versucht, unter entsprechenden Schutzmaßnahmen den Praktikumsbetrieb aufrechtzuerhalten. Parallel dazu wurden aber auch digitale Lernangebote im Rahmen des pharmazeutischen Curriculums entwickelt. Im Folgenden werden digitale Lehrangebote als Praxisbeispiele näher erläutert und in das Spektrum der oben beschriebenen Lernszenarien eingeordnet (Abb. 1).

### Audio-Podcasts zur Leistungsbeurteilung einer Praktikumsstation im Praktikum Klinische Chemie

Das Praktikum Klinische Chemie ist an der Universität Greifswald im 5. Fachsemester angesiedelt. Dort werden Methoden zur Bestimmung laborchemischer Parameter in der klinischen Praxis vermittelt. An verschiedenen Stationen führen die Studierenden eigene Untersuchungen durch. Eine dieser Stationen beschäftigt sich mit diagnostischen Systemen wie Urinteststreifen, Blutzuckermessgeräten, Schwangerschaftstests, SARS-CoV-2-Testsystemen und Pulsoximetern. Es ist die praktische Aufgabe der Studierenden, diese Testsysteme zu nutzen, um teils eigens dafür hergestellte Urinproben (Urinteststreifen, Schwangerschaftstest) zu untersuchen oder an sich selbst anzuwenden (Pulsoximeter). Die Durchführung der Tests und die Interpretation der Ergebnisse werden von den Studierenden in einem Protokoll zusammengefasst.

Alternativ zur Erstellung von Protokollen wurde für die genannte Station wahlweise für die Nutzung von Urinteststreifen oder die Anwendung eines Pulsoximeters ein Audio-Podcast als Lernmethode eingeführt [5]. Hier handelt es sich am ehesten um ein digitalisiertes Lernelement, mit dem die Lehrveranstaltung angereichert wird und das dem Lernszenario Interaktion und Kollaboration zugeordnet werden kann. Als Anforderungen sollten die Audio-Podcasts alle Informationen enthalten, die für die Protokolle notwendig sind, und nicht länger als 3–5 Minuten sein.

In einer nachfolgenden Evaluation gaben mehr als 2 Drittel der Studierenden an, dass sie die Abwechslung gegenüber einem Protokoll schätzten, dass die Kreativität herausgefordert wird, kommunikative Fähigkeiten gestärkt werden und die Medienkompetenz erhöht wird, dass die Erstellung der Podcasts als Gruppenarbeit gegenüber einer Einzelarbeit bevorzugt wird und dadurch auch die organisatorischen Fähigkeiten der Studierenden unterstützt werden und es möglich ist, die Vorstellung der einzelnen Studierenden einzubringen. Außerdem waren sich alle Studierenden darin einig, dass sich die angebotenen Themen gut für die Erstellung von Podcasts eigneten.

### Nutzung eines digitalen Analyseinstruments zur Durchführung von Medikationsanalysen

Die Durchführung einer Medikationsanalyse ist inzwischen eine durch die Krankenkassen extra honorierte pharmazeutische Dienstleistung in öffentlichen Apotheken. Daher sollten die Studierenden schon im Studium mit diesem Prozess vertraut gemacht werden. An der Universität Düsseldorf wurde ein digitales Analyseinstrument zur Unterstützung der Durchführung einer Medikationsanalyse im Rahmen eines Seminars der klinischen Pharmazie eingeführt. Die Studierenden erhielten eine Schulung zur Nutzung des digitalen Analyseinstruments, in der diese auch selbstständig Informationen von Patienten eingaben und das Ergebnis gemeinsam diskutierten.

Im Rahmen einer randomisierten, kontrollierten Studie wurde der Nutzen des digitalen Analyseinstruments zur Unterstützung der Medikationsanalyse untersucht [6]. Dabei erhielten die Studierenden Patientenfälle und mussten mit diesen in einem definierten begrenzten Zeitraum Medikationsanalysen durchführen. Eine der zufällig zugeteilten Studierendengruppen führte die Medikationsanalyse ohne Nutzung des digitalen Analyseinstrumentes durch, die andere Gruppe nutzte dagegen das digitale Analyseinstrument. Die Leistung der Studierenden wurde im Rahmen eines objektiven, strukturierten, klinischen Assessments geprüft. Insgesamt zeigte sich, dass diejenige Studierendengruppe, die das digitale Analyseinstrument zur Verfügung hatte, eine bessere Leistung erzielen konnte als die Studierendengruppe, die das digitale Analysentool nicht nutzen konnte. Darüber hinaus war sich nur die Studierendengruppe, welche das digitale Analyseinstrument nutzte, darüber einig, dass sie sich bei der Durchführung der Medikationsanalyse und dem darauffolgenden Gespräch mit dem Arzt sicher gefühlt haben. Beide Gruppen hingegen hielten die Anwendung des digitalen Analyseinstruments für nützlich sowohl zur Durchführung der Medikationsanalyse als auch als Lerninstrument für das Fach Klinische Pharmazie.

### Blended Learning zur Vermittlung von Grundlagen der klinischen Pharmazie

**Abb. 2:**
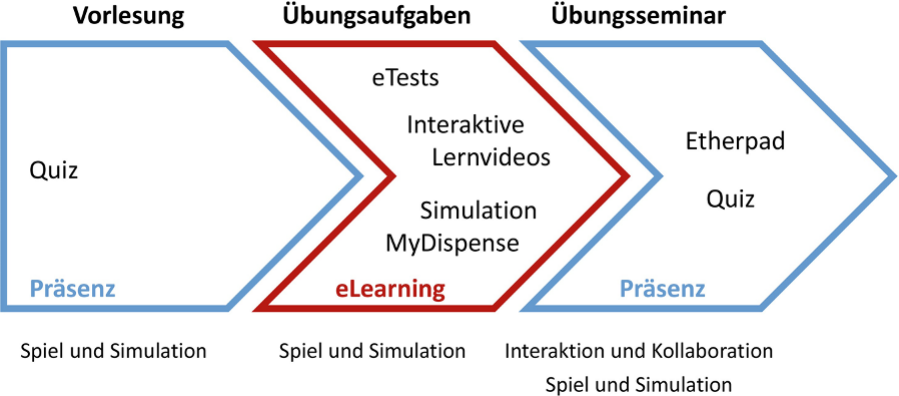
Blended-Learning-Struktur des Lehrveranstaltungskonzeptes „Grundlagen Klinische Pharmazie“ an der Universität Greifswald mit Einbindung von Lernelementen, Lernformaten und Lernszenarien. Eigene Abbildung

An der Universität Greifswald werden Lerninhalte der klinischen Pharmazie im 7. und 8. Fachsemester des Studiengangs Pharmazie vermittelt. Im 7. Semester werden im Rahmen einer Grundlagenveranstaltung wochenweise verschiedene Themen der klinischen Pharmazie erarbeitet. Die Struktur der Lehrveranstaltung folgt dem Lernkonzept des Blended Learning (Abb. 2). Die Studierenden erfahren in einer Präsenzvorlesung mit integrierten Quizfragen zunächst grundlegende Wissensinhalte. Diese sollen in einer sich anschließenden Lernphase mittels Testfragen, interaktiver Lernvideos und Simulationen, die über die Lernplattform elektronisch zur Verfügung gestellt werden, wiederholt, verinnerlicht und verstanden werden. In einem sich daran anschließenden Übungsseminar werden Wissensinhalte vertieft und auf neue Situationen angewendet. Dabei bearbeiten die Studierenden Fragestellungen innerhalb von Seminargruppen. Hierfür stehen Etherpads, d. h. webbasierte Texteditoren, als kollaborative Arbeitshilfen zur Verfügung. Die Ergebnisse der Gruppenarbeit werden dann in einem abschließenden Quiz gemeinsam besprochen. Die Quizfragen dienen dabei der Prüfung, ob die Inhalte verstanden wurden, und stellen Ausgangspunkte für weitergehende Diskussionen dar.

Aufgrund des Blended-Learning-Charakters ist die Struktur der Lehrveranstaltung dem Lernszenario Integration zuzuordnen. Die Lehrveranstaltung enthält aber auch in allen Lernphasen Elemente des Lernszenarios Spielen und Simulation sowie in der dritten Phase im Rahmen des Übungsseminars Elemente des Lernszenarios Interaktion und Kollaboration. Mehr als 3 Viertel der Studierenden konnten anhand der E‑Learning-Übungen Lerninhalte erinnern und wiederholen bzw. ihr Verständnis überprüfen. Die Studierenden empfanden die Übungsaufgaben mit den Lerninhalten abgestimmt und im Umfang angemessen. Die Quizze wurden ebenfalls als geeignetes Element empfunden.

### Virtuelle Lehre am Krankenbett

**Abb. 3:**
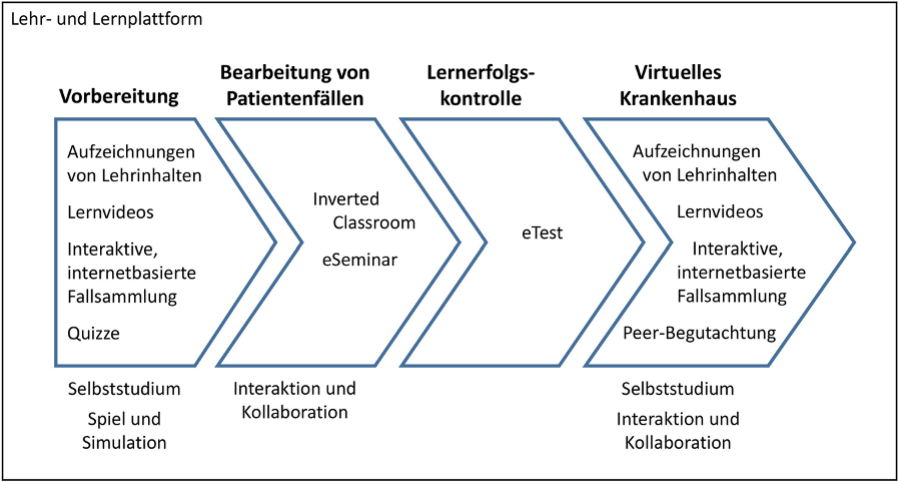
Struktur des Online-Seminars zur virtuellen Lehre am Krankenbett an der Universität München mit Einbindung von Lernelementen, Lernformaten und Lernszenarien. Eigene Abbildung

An der Universität München wird für Studierende der Pharmazie im Rahmen des Seminars Klinische Pharmazie seit 2014 Lehre am Krankenbett angeboten. Im Rahmen der SARS-CoV-2-Pandemie wurde ein Konzept der virtuellen Lehre am Krankenbett entwickelt [7]. Das virtuelle Konzept wurde als ausschließlicher Online-Kurs auf einer Lernplattform realisiert (Abb. 3). Der Kurs bestand aus unterschiedlichen Phasen. Die erste Phase diente der Wiederholung relevanter Lerninhalte. Es war hier die Aufgabe der Studierenden, anhand eines Fallbeispiels aus einer interaktiven, internetbasierten Fallsammlung eine Arzneimittelanamnese im Rahmen der Aufnahme des Patienten durchzuführen. Es wurden den Studierenden dazu Lerninhalte aus vorangegangenen Lehrveranstaltungen zu den Themen Kommunikation, Arzneimittelinformation und Pharmakologie zur Verfügung gestellt. Die zweite Lernphase beinhaltete die erste aktive Übung. In dieser mussten die Studierenden in Kleingruppenarbeit für 2 fiktive Patienten jeweils eine Medikationsanalyse durchführen. Alle notwendigen Unterlagen wurden den Studierenden zur Verfügung gestellt, außerdem erhielten sie Informationen zu relevanten Informationsquellen und Analyseinstrumenten.

In der dritten Lernphase erhielten die Studierenden Zugang zu einer virtuellen Krankenhausstation mit mehreren fiktiven Patienten. Diese wurde durch Screenshots von Einträgen fiktiver Patienten aus der klinischen Dokumentationssoftware des Krankenhauses und der Bereitstellung von fiktiven Patientenunterlagen (Auszüge aus der Patientenakte, Arztbriefe, Laborwerte) realisiert. Es war in dieser Phase die Aufgabe der Studierenden, die Unterlagen der Patienten zu sichten und für einen dieser Patienten einen pharmazeutischen Betreuungsplan zu entwickeln. Die Bearbeitung der Aufgaben während der Lernphasen konnten sich die Studierenden selbst einteilen, sie mussten dabei lediglich eine Abgabefrist beachten. Die Bewertung der Ergebnisse der Bearbeitung der Aufgaben erfolgte individuell durch die Dozentin. Im Rahmen der dritten Lernphase erhielten die Studierenden zudem eine Anfrage zur Medikation einer bestimmten Patientin durch eine behandelnde Ärztin. Diese Anfrage mussten die Studierenden schriftlich beantworten. Die Bewertung dieser Arzneimittelinformation erfolgte anhand eines kollaborativen Peer-Assessments. Als Motivationsanreiz wurden Badges (digitale Abzeichen) für Leistungen mit unterschiedlichen Schwierigkeitsgraden vergeben. Besonders positiv wurden von den Studierenden die selbstständige, individuelle Arbeit und das persönliche Feedback bewertet. Die Struktur dieses Online-Kurses könnte besonders für Standorte eine gute Anregung sein, eine am Patienten nahe Lehre zu vermitteln, die keine Möglichkeit für einen direkten Patientenkontakt haben.

## MyDispense – spielähnliche Simulation zur Abgabe von Arzneimitteln

**Abb. 4:**
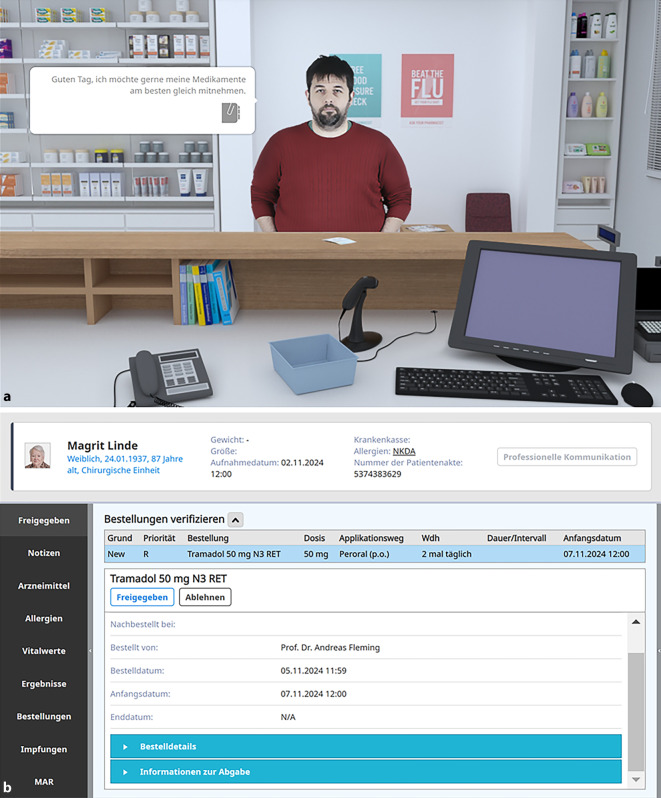
Screenshots von MyDispense-Übungsaufgaben **a** zur Abgabe von Arzneimitteln in der öffentlichen Apotheke und **b** zur Bewertung einer Arzneimittelanforderung von einer Station eines Krankenhauses. Bei den in den Screenshots dargestellten Personen handelt es sich um fiktive Patienten. MyDispense Version 7.3.29 ger

Die Simulationssoftware MyDispense wurde an der Monash University, Australien, entwickelt [8]. Die Software erschafft einen virtuellen Raum, in dem Arzneimittel abgegeben werden. Der Abgabeprozess ist mit verschiedensten Aufgaben der Bewertung der Abgabesituation und der Übermittlung relevanter Informationen zur korrekten Anwendung des Arzneimittels verknüpft. Die Abgabesituation kann sich in der öffentlichen Apotheke, in der Ambulanz eines Krankenhauses oder im Rahmen einer Anfrage einer Station befinden (Abb. 4). Seit 2017 wurde an der Universität Greifswald die Software zusammen mit den Entwicklern aus Australien an die Bedingungen des Arzneimittelverkehrs in Deutschland angepasst [9]. Dazu mussten die rechtlichen Rahmenbedingungen eingebracht werden (Aussehen und Anforderungen an die Verschreibungsformulare), ein für Deutschland repräsentatives Arzneimittelsortiment aufgebaut und die Software mit verschiedenen Arten von Personen bevölkert werden (Verschreibende verschiedener Fachrichtungen, Patienten mit verschiedensten Eigenschaften). Außerdem wurden alle für die Studierenden in der Nutzungsoberfläche sichtbaren Informationen ins Deutsche übersetzt. Seit 2019 steht so eine MyDispense-Version für Deutschland bereit.

Mit MyDispense Deutschland können verschiedenste Übungen zur Abgabe von Arzneimitteln erstellt werden. Das Spektrum reicht von der rein formalen Prüfung von Verschreibungen, der Abgabe von Arzneimitteln auf Verschreibung, der Abgabe von Arzneimitteln im Rahmen der Selbstmedikation oder der Bewertung einer Anforderung eines Arzneimittels für einen Patienten einer Station eines Krankenhauses. Während der Bearbeitung der Übung lernen die Studierenden verschiedene Fähigkeiten, die für die Bewertung der Abgabesituation wichtig sind. Dazu gehören die Nutzung relevanter Fachliteratur, die Erfassung von Angaben zum Patienten im Apothekenmanagementsystem und Gewinnung von Information im Gespräch mit dem Patienten und seinem behandelnden Arzt. So können die verschiedensten Abgabeszenarien in unterschiedlichsten Komplexitäten generiert und durchgespielt werden. An der Universität Greifswald sind solche Übungsaufgaben integraler Bestandteil der Lehrveranstaltungen der klinischen Pharmazie. Die Studierenden werden anhand eines Leitfadens der Bundesapothekerkammer zur Abgabe eines Arzneimittels auf Verschreibung schrittweise an diesen Prozess herangeführt und können mit jedem Schritt die Fähigkeiten der vorangegangenen Schritte wiederholen und trainieren. Nach jeder Übung erhalten die Studierenden ein automatisiertes Feedback, das sie für die unbegrenzte Wiederholung dieser und der nachfolgenden Übungen berücksichtigen können. Jeder Prozessschritt wird mit einer Assessment-Übung abgeschlossen, die durch den Dozenten bewertet wird.

Da auch an anderen pharmazeutischen Standorten in Deutschland, in Österreich und der Schweiz sowie vonseiten von Apothekerkammern Interesse an der Nutzung von MyDispense bestand, wurde das MyDispense Netzwerk Deutschland gegründet. Kern des Netzwerks ist eine Austauschplattform für MyDispense-Übungsaufgaben und verbundenen Lernmaterialen. Zudem findet ein persönlicher Austausch zwischen den Hochschullehrenden, die MyDispense nutzen, per Online-Konferenz 4‑mal im Jahr statt.

## Einbindung künstlicher Intelligenz in digitale Lehrformate in der Pharmazie

Die Nutzung von künstlicher Intelligenz im Rahmen der Lehre in der Pharmazie wird aktuell kontrovers diskutiert. Das Berufsbild des Apothekers wird mit seinen zunehmend komplexen Dienstleistungen immer anspruchsvoller. Gerade bei der Durchführung von Medikationsanalysen, der Auswahl und der Abgabe von Arzneimitteln, der Überwachung von unerwünschten Arzneimittelwirkungen und der Identifizierung und Bewertung von Arzneimittelinteraktionen kann die Unterstützung durch künstliche Intelligenz hilfreich sein. Für eine sinnvolle und zuverlässige Anwendung sind aber einige Voraussetzungen zu erfüllen: So ist es notwendig, die Fragestellung korrekt zu formulieren (Prompting) und alle relevanten Parameter zu berücksichtigen. Außerdem müssen die den Algorithmen zur Verfügung stehenden Daten transparent, von hoher Qualität und aktuell sein. Kuratierte Datensätze, mit denen die Sprachmodelle der künstlichen Intelligenz entsprechend ihres Einsatzzweckes trainiert werden, gewährleisten eine hohe Qualität [10].

Für den Einsatz künstlicher Intelligenz in der Lehre besteht die Gefahr, dass insbesondere die Entwicklung von kritischem Denken und die Fähigkeit, eigene Entscheidungen zu treffen, behindert werden [11, 12]. Mit sorgfältiger Planung könnte künstliche Intelligenz sinnvoll etwa die Strukturierung von Lehrveranstaltungen, die Erstellung von klinischen Patientenfällen, die Formulierung von Arbeitsaufträgen oder die Erarbeitung von Prüfungsfragen unterstützen. Darüber hinaus sollte der kritische Umgang mit künstlicher Intelligenz vermittelt werden, indem zum Beispiel klinische Fragestellungen durch künstliche Intelligenz beantwortet werden und diese Antworten kritisch hinterfragt und interpretiert werden [13].

## Fazit

Es stehen vielfältige Lernelemente und Lernformate zur Verfügung, die in den unterschiedlichsten Lernszenarien in unterschiedlichem Ausmaß und in unterschiedlicher Komplexität angewendet und teilweise kombiniert werden können. Durch die ausgewählten Anwendungsbeispiele konnte die große Flexibilität in der Nutzung digitaler Lernmethoden dargestellt werden. Die überwiegende Mehrheit der Studierenden steht digitalen Lernmethoden positiv gegenüber. Ein wesentlicher Aspekt, der die Einführung digitaler Lernmethoden behindern kann, ist der relativ hohe zeitliche und personelle Aufwand. Dieser entsteht nicht nur auf der Seite der Lehrenden, die entsprechende Konzepte entwickeln und realisieren müssen, sondern kann auch zu einer Überbelastung der Studierenden führen, wenn der zeitliche Aufwand der Studierenden für konkurrierende Lehrveranstaltungen nicht berücksichtigt wird. Daher sollten diese Aspekte bei der Planung digitaler oder digitalisierter Lehrveranstaltungen mit bedacht und den Studierenden gegenüber vermittelt werden. Zeit- und Personalaufwand könnten durch einen stärkeren Austausch und eine engere Vernetzung zwischen den Standorten reduziert werden. Ein erster Schritt hierfür wurde durch die Gründung des MyDispense Netzwerks Deutschland gemacht. Grundsätzlich lässt die Approbationsordnung für Apotheker begleitende digitale Lehrformate für Seminare und praktische Lehrveranstaltungen zu. Auch Vorlesungen können in digitaler Form durchgeführt werden. Um digitale Lernangebote für die Nutzung im Rahmen von Lehrveranstaltungen der Pharmazie aber weiter zu entwickeln, bedarf es tiefergehender Veränderungen im Umfang der Lehrinhalte sowie der Struktur des Pharmaziestudiums. Eine kluge Novellierung der Approbationsordnung könnte hierfür geeignete Rahmenbedingungen schaffen.
